# Pathologic and Neuropathologic Study of a Case of COVID-19

**DOI:** 10.31662/jmaj.2021-0178

**Published:** 2021-12-24

**Authors:** Masashi Mizutani, Yuji Nakayama, Yuji Saitoh, Hajime Ariga, Takako Enokida, Tasuku Ishihara, Terunori Sano, Yuichiro Hirata, Harutaka Katano, Tadaki Suzuki, Masaki Takao

**Affiliations:** 1 Department of Laboratory Medicine, National Center of Neurology and Psychiatry, Tokyo, Japan; 2 Department of Pathology, Keio University School of Medicine, Tokyo, Japan; 3 Department of Neurology, National Center of Neurology and Psychiatry, Tokyo, Japan; 4 Department of General Internal Medicine, National Center of Neurology and Psychiatry, Tokyo, Japan; 5 Department of Psychiatry, National Center of Neurology and Psychiatry, Tokyo, Japan; 6 Department of Pathology, National Institute of Infectious Diseases, Tokyo, Japan

**Keywords:** COVID-19, autopsy, neuropathology, brain, spleen, immunohistochemistry, electron microscopy, Japanese

## Abstract

A 68-year-old woman with a history of schizophrenia developed coronavirus disease (COVID)-19 and was transferred to our hospital. Despite treatment, she died of respiratory failure 16 days after the onset. At the time of autopsy, polymerase chain reaction (PCR) for severe acute respiratory syndrome coronavirus 2 (SARS-CoV-2) RNA using swabs from the nasopharynx and the lung was positive; however, the cerebrospinal fluid was negative. An autopsy showed diffuse alveolar damage and recent multiple cerebral infarcts. Acute splenitis was observed with thrombi adhering to the vascular endothelium in areas of severe neutrophilic infiltration. Immunohistochemistry using an antibody against the SARS-CoV-2 nucleocapsid showed immunoreactivity along the hyaline membrane of the lung; however, the antibody showed no immunoreactivity in the medulla, the thalamus, the frontal lobe, and the pituitary. Future pathologic studies should clarify the mechanisms involved in a variety of clinical and pathological changes related to COVID-19.

## Introduction

Coronavirus disease (COVID)-19, a disease caused by severe acute respiratory syndrome coronavirus 2 (SARS-CoV-2) infection, is characterized by fever, pneumonia, dysosmia, and dysgeusia, as well as symptoms related to the central and peripheral nervous systems. Pathological studies of COVID-19 reported that SARS-CoV-2 was detected in the central nervous system, particularly in the medulla, cerebrum, and olfactory bulb, as well as in various general organs ^(1)^. However, most autopsy studies have been conducted in western countries ^(1), (2), (3), (4)^. Here, we report an autopsy case of a Japanese individual with COVID-19, from a pathological viewpoint, using conventional, immunohistochemical, and electron microscopic methodologies.

## Case Report

**Figure 1.**
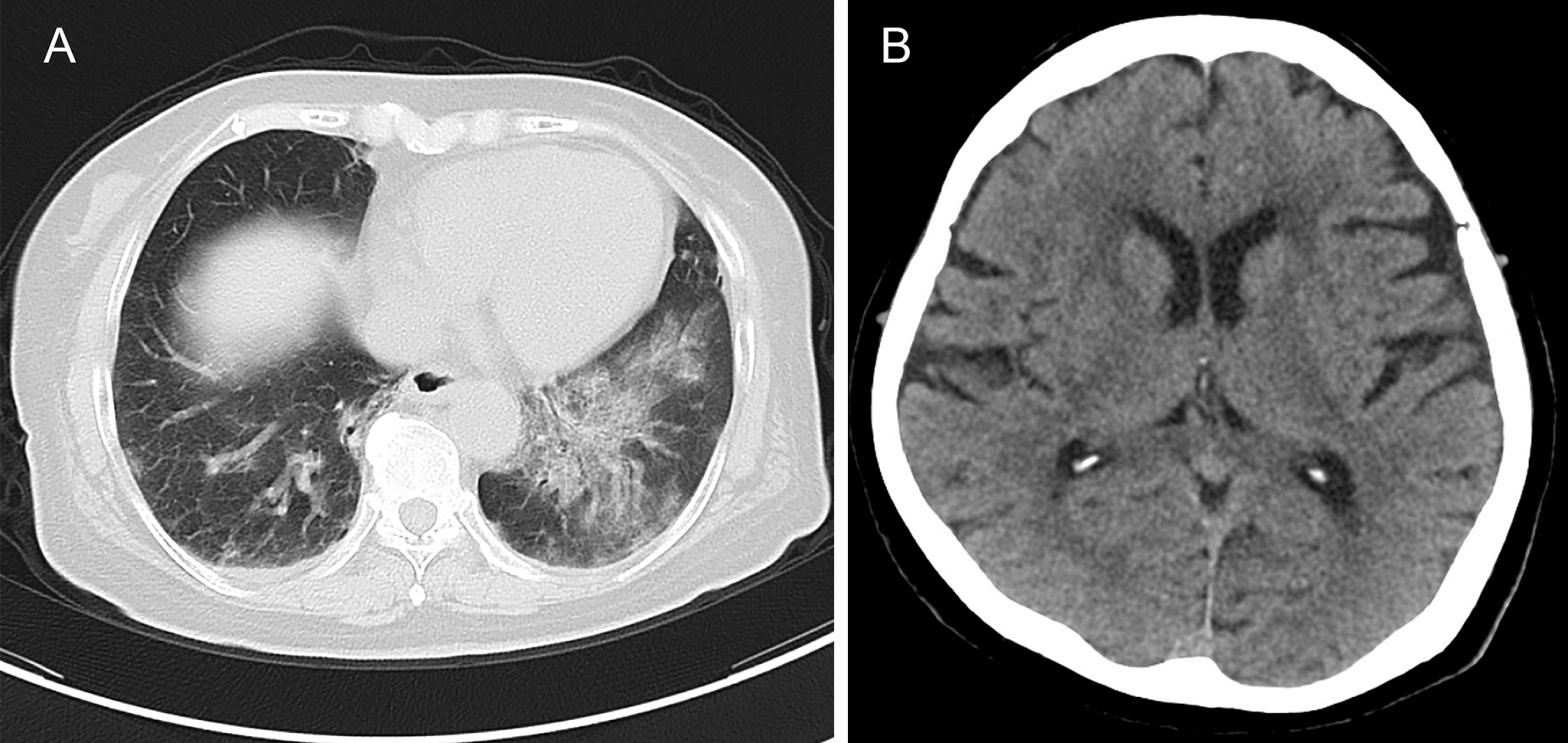
Computed tomography images of the chest and head. A) Chest computed tomography (CT) image taken on admission shows slightly left and bottom dominant ground-glass appearance and pleural fluid. B) Head CT images taken on the same day show no obvious infarction or hemorrhage.

A 68-year-old woman with schizophrenia developed COVID-19 and was transferred to our hospital. Chest computed tomographic images showed bilateral ground-glass opacities consistent with COVID-19 (Figure 1). Haloperidol and chlorpromazine were continued, and no deterioration was observed in her psychiatric conditions. She died of respiratory failure 16 days after the onset. An autopsy was performed 18 hours after her death. To avoid the risk of infection to the medical staff, pieces of each organ (heart, lungs, spleen, liver, gall bladder, pancreas, stomach, jejunum, appendix, right kidney, and fat tissue of the anterior mediastinum) were obtained and analyzed for histology. Plenty of yellowish bilateral pleural effusions were observed. Polymerase chain reaction (PCR) for SARS-CoV-2 RNA using swabs from the nasopharynx and the lung was positive; however, the cerebrospinal fluid was negative. PCR using frozen tissue samples was positive for the lung and frontal lobe but negative for the medulla and olfactory bulb.

### Histopathology of general organs

**Figure 2.**
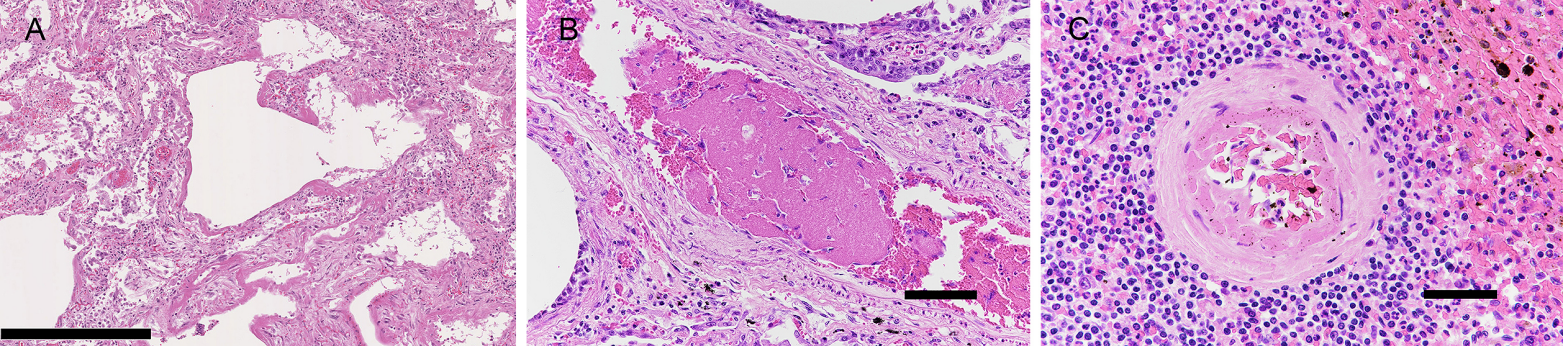
Photomicrographs of the lung and spleen. A) The lung shows diffuse alveolar damage in the exudative to proliferative phases and remodeling of the alveolar wall. B) A small number of fibrin thrombi are present in the small- and medium-sized vessels of the lung. C) The spleen shows acute splenitis. A thrombus adhering to the injured endothelium is present in areas with a high degree of neutrophil infiltration. Scale bar: A: 500 μm; B: 100 μm; C: 50 μm.

The lungs contained inflammatory cell infiltrates, including macrophages, lymphocytes, and neutrophils in the alveolar walls. There was hyaline membrane formation along the alveolar wall and thickening of the alveolar septa with fibrosis (Figure 2A). A few fibrin thrombi were observed in small vessels (Figure 2B). The spleen contained occasional thrombi adhering to the injured endothelium in proportion with the neutrophil infiltration (Figure 2C). Acute tubular necrosis was present in the kidney. Neutrophil infiltration in the sinusoids and regenerative changes of hepatocytes were observed in the liver.

### Neuropathology

**Figure 3.**
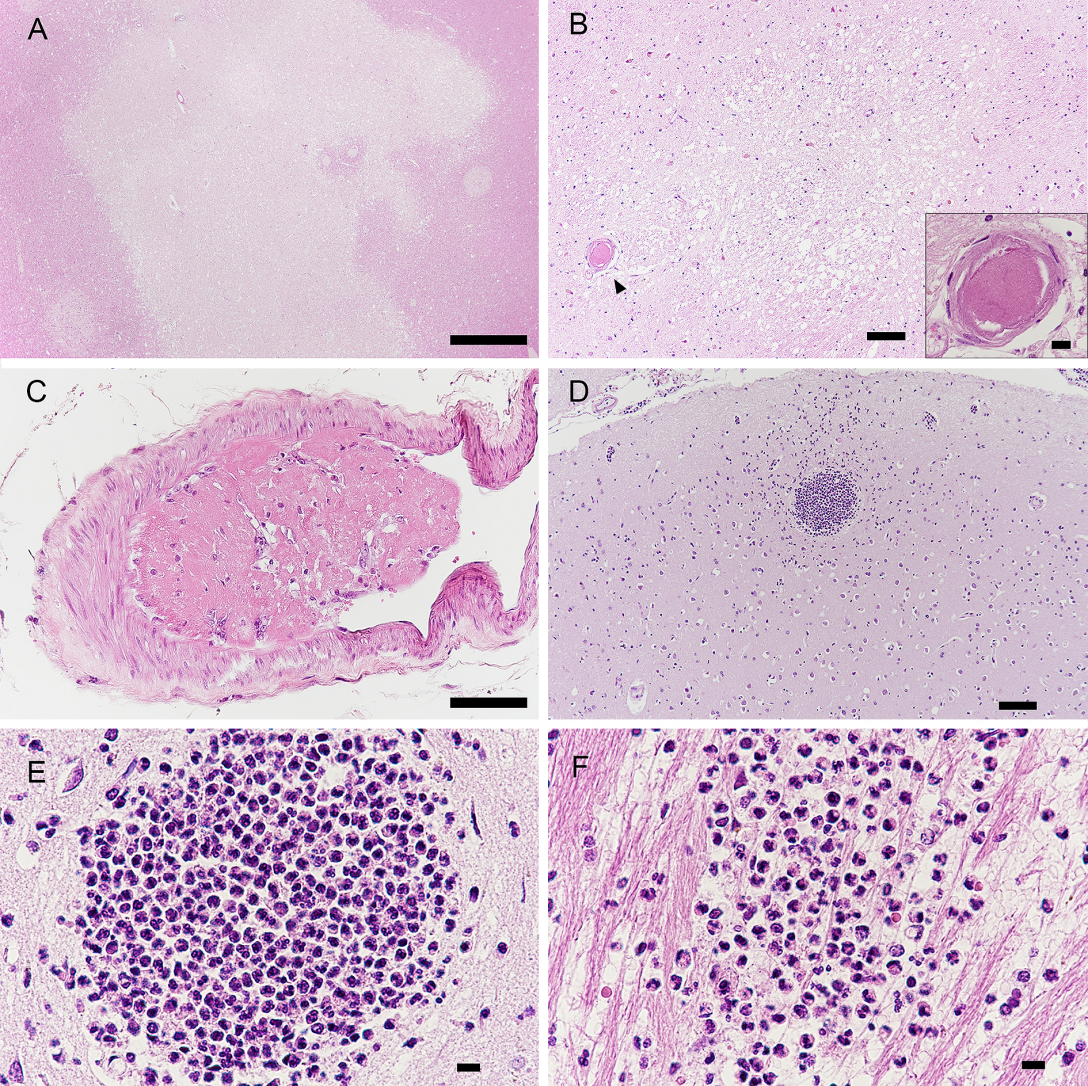
Photomicrographs of the cerebrum. A) An infarct in the white matter of the parietal lobe. B) A small infarction is observed in the thalamus. Inset; close to the lesion, a fibrin thrombus is present in a vessel (arrowhead), as shown in the inset. C) An organized thrombus in the leptomeningeal vessel of the frontal lobe. The thrombi shown in B and C are similar to those shown in Fig. 2B. D, E) Clustered neutrophils in the left parietal cortex and F) internal capsule. Scale bar: A: 1000 μm; B, C, D: 100 μm; B (inset), E, F: 10 μm.

The brain weighed 1202 g and showed no atrophy. Mild atherosclerosis was seen in the basilar artery. Microscopic examination revealed acute cerebral infarcts in the left middle frontal cortex, the precentral cortex, the thalamus, and the cerebral white matter (Figure 3A, B and C). There were fibrin thrombi and organized thrombi in the parenchymal and leptomeningeal small vessels of the cerebrum. Micro abscesses were observed in the parietal cortex and internal capsule (Figure 3D, 3E and 3F).

### Immunohistochemistry and electron microscopy

**Figure 4.**
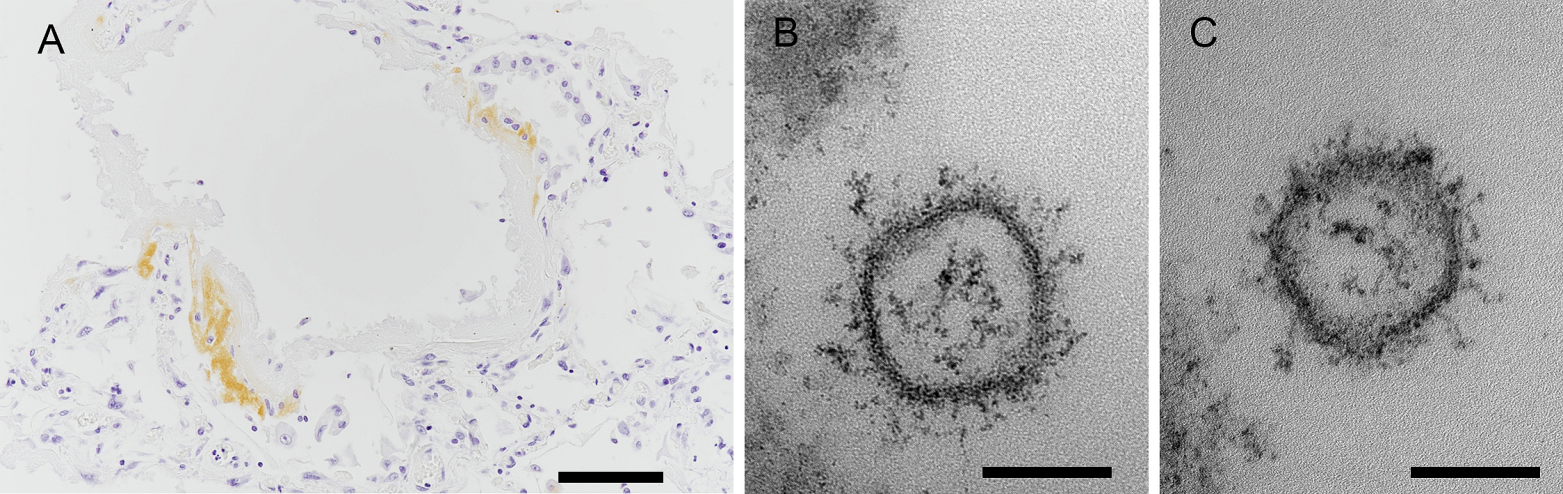
Immunohistochemistry and electron micrograms. A) Immunohistochemistry of the right lung shows that hyaline membranes are positive for the SARS-CoV-2 nucleocapsid. B, C) Small vesicular structures suspected to be SARS-CoV-2 observed by electron microscopy in the extracellular space of the lung. Scale bar: A: 100 μm; B, C: 100 nm.

There was immunoreactivity on the hyaline membrane of the lung using an antibody for the SARS-CoV-2 nucleocapsid (40143-R001; Sino Biological, Eschborn, Germany) (Figure 4A) ^(4)^. However, the antibody showed no immunoreactivity in the medulla, the thalamus, the frontal cortex, and the pituitary. Electron micrograms (EM) showed small vesicular structures suggesting SARS-CoV-2 virions in the lung (Figure 4B, C). Viral particles were not detected in the olfactory bulb or the frontal cortex by EM.

## Discussion

Acute splenitis occurs in ~20% of COVID-19 cases and is more common than in SARS-CoV-1 cases ^(5)^. Neutrophils are thought to have important roles in the pathogenesis of COVID-19 ^(6)^. Microthrombus formation associated with neutrophil extracellular traps, so-called immunothrombosis, was reported in the lungs of COVID-19 cases ^(6)^. Indeed, the present case had frequent microthrombi in areas with a high degree of neutrophil infiltration. Interestingly, a patient with schizophrenia who developed COVID-19 had an increased mortality rate, which might have been related to immune dysregulation ^(7)^.

Although microthrombi in the cerebral vessels were present in our case, we could not conclude whether they were caused by COVID-19. It was difficult to interpret the discrepancy between PCR and immunohistochemistry of the frontal cortex. Since the SARS-CoV-2 antibody is reliable, contamination of samples with parenchymal blood might have occurred. Since EM analysis was available for limited anatomical areas, future studies should clarify the distribution of virions in the brain. Nath et al. reported microvascular injuries in the brain of a patient with COVID-19 ^(8)^; however, there was no clear evidence of microvascular injuries in the brain of the present case. Indeed, our case did not show any neurological symptoms during the clinical course. Although micro abscesses are not specific for COVID-19, they may be associated with the dissemination of the virus.

We report the general and neuropathologic findings of an autopsy case of a Japanese patient with COVID-19. This study is important because of the rarity of autopsy reports, including the brain, of Japanese patients with COVID-19. Further pathologic studies may be warranted to clarify the mechanisms related to the various clinical and pathological changes of COVID-19.

## Article Information

### Conflicts of Interest

None

### Sources of Funding

This work was supported in part by a grant-in-aid from AMED grant number JP21wm0425019 (MT) and an intramural fund from the National Center of Neurology and Psychiatry (3-8, MT).

### Acknowledgement

We acknowledge Dr. Takamasa Noda, Dr. Naoko Satake, Dr. Takuma Inagawa, and Dr. Atsushi Unuma for providing the clinical treatment. We also thank Mr. Ryo Wakabayashi, Ms. Emi Usukura, Ms. Ayako Sato, and Mr. Mitsuhiro Sakamoto for their technical assistance with the safe handling of the autopsy and immunohistochemical study. We thank J. Ludovic Croxford, PhD, from Edanz (https://jp.edanz.com/ac) for editing a draft of this manuscript.

### Author Contributions

All authors meet the ICMJE authorship criteria.

MM, YN: acquisition of data, analysis and interpretation of data, and drafting of the manuscript; YS, HA, TE: were in charge of the patient in our hospital; TS, TI, YH, HK, TS: acquisition and analysis and interpretation of data; MT: conception and design of the study, analysis and interpretation of data, and drafting or revision of the manuscript.

### Informed Consent

Written informed consent was obtained from the next-of-kin of the patient for the autopsy and research. The brain autopsy and brain bank were approved by institutional review boards．
